# Lectures on Diseases of the Nervous System

**Published:** 1876-01

**Authors:** 


### Books Reviewed.

*Lecture on Diseases of the Nervous System.* By JEROME K. BAUDNY, M. D., Professor of Psychological Medicine, etc., in the Missouri Medical College. Reported by V. BIART, M. D., Revised and Edited by the Author. Philadelphia: J. B. Lippincott & Co., 1876. Buffalo: Martin Taylor.

This work does not pretend to present to the profession any essentially new or original ideas, it is, however, an excellent condensation of the more generally accepted ideas upon the Diseases of the Nervous System, and is well adapted to the wants of the student or general practitioner. The lectures are forty in number, and are arranged in a systematic order.

The various forms of cerebral hyperæmia and anæmia are first considered. The inflammations of the meningies are next considered; following are six lectures devoted to the subject of insanity. Apoplexy and Cerebral Hemorrhage are treated in two lectures. In the portion devoted to treatment of the subject of blood letting is discussed and condemned.

In the consideration of the medico-legal relations of epilepsy the author goes very fully into the subject, and has made this a very interesting and instructive portion of his work. The concluding chapters of the work are upon diphtheritic paralysis, various infantile paralyses, aphasia, syphilitic nervous affections, neuralgia and glosso-labio-laryngeal paralysis.

The lecture upon syphilitic nervous affections is, without going too fully into detail, a very full exposition of what may be suspected in syphilitic patients subject to nervous disorders of any kind. The lecturer wisely cautions his hearers not to place every ill-defined disorder to the credit of syphilis, but nevertheless tells them what they are to fear in making a diagnosis, and what to do when the diagnosis is made.

